# Competition and co-association, but not phosphorous availability, shape the benefits of phosphate-solubilizing root bacteria for maize (*Zea mays*)

**DOI:** 10.1099/acmi.0.000543.v3

**Published:** 2023-12-08

**Authors:** Joseph Williamson, Andrew Charles Matthews, Ben Raymond

**Affiliations:** ^1^​ Department of Life Sciences, Silwood Park campus, Imperial College London, Ascot, SL5 7PY, UK; ^2^​ Centre for Biodiversity and Environment Research, Department of Genetics, University College London, Gower St, London, WC1E 6BT, UK; ^3^​ College of Life and Environmental Sciences, Penryn campus, University of Exeter, Penryn, TR10 9FE, UK

**Keywords:** bacterial community assembly, plant growth promotion, PGPR, plant microcosm, plant mutualists, rhizobacteria

## Abstract

Predicting the conditions under which rhizobacteria benefit plant growth remains challenging. Here we tested the hypothesis that benefits from inoculation with phosphate-solubilizing rhizobacteria will depend upon two environmental conditions: phosphate availability and competition between bacteria. We used maize-associated rhizobacteria with varying phosphate solubilization ability in experiments in soil, sterilized soil and gnotobiotic microcosms under conditions of varying orthophosphate availability, while we manipulated the intensity of competition by varying the number of isolates in plant inocula. Growth promotion by microbes did not depend on phosphate availability but was affected by interactions between inoculants: the beneficial effects of one *Serratia* isolate were only detectable when plants were inoculated with a single strain and the beneficial effects of a competition-sensitive *Rhizobium* was only detectable in sterilized soil or in microcosms inoculated with single strains. Moreover, microcosm experiments suggested that facilitation of a parasitic isolate, not competitive interactions between bacteria, prevented plants from gaining benefits from a potential mutualist. Competition and facilitation affected colonization of plants in microcosms but growth promotion by *Serratia* was more affected by inoculation treatment than culturable densities on roots. Experimental manipulation of seed inocula can reveal whether plant growth stimulation is robust with respect to competition, as well as the ecological strategies of different rhizobacteria. From an applied perspective, phosphate solubilization may not provide the mechanism for bacterial growth promotion but may indicate mutualistic potential due to phylogenetic associations. Importantly, benefits to plants are vulnerable to interactions between rhizobacteria and may not persist in mixed inoculations.

## Data Summary

Raw experimental data (plant growth and bacterial counts) and analysis scripts are available on Zenodo doi:10.5281/zenodo.7418377. GenBank accession numbers for sequences of focal isolates (listed in Table 1) are: ON210287, ON210286, ON210288 and ON210289.

**Table 1.** A table showing the genus of the strains used in the *Zea* mesocosm experiment. Strains were identified using 16S ribosomal amplicon sequencing. P-solubilizing phenotypes were scored by diameter of zone of clearance in spot cultures on Pikovskaya media; both P solubilization and IAA phenotypes were scored in a previous study [36]. All strains were isolated from *Zea mays* roots

Strain code	Genus and isolate	P-Solubilizing phenotype	IAA phenotype	GenBank accession
**W**	* Rahnella* r8Za	++++	*+*	ON210287
**X**	* Serratia* r1Zb	+++	−	ON210286
**Y**	* Rhizobium* r2Za	−	*+*	ON210288
**Z**	* Stenotrophomonas* r19Za	−	−	ON210289

ATP, adenosine tri-phosphate; GLM, generalized linear models; IAA, indole-3-acetic acid; LB, lysogeny broth; P, Phosphorous; PGPR, plant growth-promoting rhizobacteria.

**Impact Statement**

The solubility of phosphate in soil, rather than the total concentration, is often a key limiter of crop growth. Plant inoculation with microbes capable of phosphate solubilization is one means of increasing the availability of phosphate in soil without using additional fertilizer input. Nevertheless, field tests of plant inoculants have often had mixed results. Important questions are whether or not these microbes are effectively solubilizing phosphate for the benefit of plants and whether competitive interactions among bacteria (for example, prevention of effective root colonization) can limit plant growth promotion. Here, we show that phosphate-solubilizing bacteria can promote plant growth irrespective of the availability of phosphate, suggesting that another mechanism unrelated to phosphate is stimulating plant growth. We also showed that competitive and mutualistic interactions between microbes can determine growth promotion, indicating that microbe–microbe interactions as well as plant–microbe interactions are a key consideration for the use of microbial inoculants.

## Introduction

Bacteria play a vital role in the soil phosphorous (P) cycle and are important in increasing the availability of P for plant roots [1]. The complexities of this role and the relative magnitude of the bacterial contribution to P uptake by plants have been subjects of scientific inquiry for many years [2, 3]. Understanding the interaction between micro-organisms and roots, particularly with regard to P cycling, could have widespread benefits for agriculture [4]. Phosphate is used in a variety of essential processes within plants, such as the synthesis of adenosine tri-phosphate (ATP), as a signal molecule in metabolic processes and as an essential component of nucleic acids [5]. Due to the low mobility of P in soil, it can often be a limiting factor of plant growth, particularly in tropical and nutrient-poor regions [6]. Typically, commercial agriculture uses bulk chemical fertilizers to overcome nutrient deficiency in nitrogen, phosphorous and potassium. While nitrogen is fixed into usable forms using the Haber–Bosch process, phosphate and potassium must be extracted from mineral sources. Approximately 75 % of the world’s mineral P is located in Morocco and the Western Sahara [7]. Finite stocks and the increasing costs of mineral P are driving research into alleviating P deficiency in agricultural soils using microbial solutions [8].

A small amount of soil P is present in a form that can be directly taken up by plants. This P is known as orthophosphate and is taken up by transporters located in the root epidermis [9]. Plants can increase P uptake in orthophosphate-deficient soils using several mechanisms. Firstly, plants increase the surface area that they have in contact with the soil, either by allocating more resources to root system growth, by increasing root hairiness or by changing root architecture [10–12]. These strategies give the root access to more orthophosphate through foraging in a greater volume of soil. In many orthophosphate-deficient soils, there are still high levels of insoluble P that cannot be directly taken up, as it is often bound in calcium, aluminium or ferric complexes. The excretion of organic acids from roots can desorb P from insoluble forms to orthophosphate, thus increasing uptake by plants [11, 13, 14]. Increasing the density of P transporters in the epidermis can also increase the efficacy of P acquisition [15].

Plant growth-promoting rhizobacteria (PGPR) are now used in a variety of agricultural methods, including seed inoculation pre-sowing and the application of isolates to juvenile and mature crops in the field. These methods are often used to target nitrogen deficiencies with the application of *Rhizobium* to leguminous plants, while arbuscular mycorrhizae are more commonly used to improve phosphorous uptake [16, 17]. PGPR have also been found to promote plant growth by protecting against pathogens as biocontrol agents; by producing bio-stimulants such as indole-3-acetic acid (IAA) or by ameliorating the effects of salinity stress [18–20]. Although bacteria have not yet been applied to commercial agriculture with regard to orthophosphate deficiency, their wider potential in agriculture is becoming realized, and phosphate-solubilization assays are now widely used in PGPR screening programmes [2, 21–23].

PGPR are hypothesized to be able to increase acquisition of P from soil through indirect and direct mechanisms [24]. Indirect mechanisms tend to rely on hormonal manipulation of plant physiology, causing shifts in root architecture, root hair development or relative investment in roots, all of which can increase the P foraging capability of the plant [19, 25]. These strategies are a microbiologically mediated version of the same P-foraging strategies that plants use themselves. By contrast, direct mechanisms of P solubilization rely on the biochemistry of PGPR. Specifically, they can produce protons and/or organic acids that lower environmental pH in order to unbind insoluble P from metal ions. Similarly, siderophores can remove orthophosphate from ferric ions [26], and some PGPR produce enzymes that can catalyse P solubilization. These orthophosphate-releasing pathways are relatively easy to identify in a laboratory but are hard to distinguish from plant-mediated P solubilization in field experiments, making it difficult to demonstrate the mechanistic basis of how rhizobacteria benefit plant hosts in the field [27, 28].

An ongoing issue with commercial PGPR implementation is that plant inoculation can give inconsistent effects in the field [29–31], a problem that can deter growers from investing in formulated products. The specific mechanisms by which bacteria stimulate growth are often not known, since multiple candidate physiological effects could be responsible for increased growth in controlled experiments [19]. It is therefore difficult to predict when plant inoculation with putative PGPR is going to be beneficial for growers. Fungal inoculants can provide more benefits to plants where orthophosphate is limiting but P is present in an insoluble form [32]. We hypothesize that phosphate solubilization by rhizobacteria will also be beneficial for plants when orthophosphate is limiting and there is available phosphate that could be mobilized, although we note that PGPR can increase the value of fertilizers and benefit plants in high-nutrient environments [33, 34].

An additional potentially widespread limitation of the application of PGPR is competition with other microbes that may limit the colonization of crop root material [31, 35]. There is increasing evidence that competitive interactions are important forces in structuring rhizobacterial communities [36]. Indeed, crop species can have distinct microbial communities that may vary in terms of how susceptible they are to colonization by applied PGPR [36, 37]. While it is hard to subtly manipulate levels of competition within complex bacterial communities in soil, we can increase overall levels of competition by increasing the numbers of isolates in each plant inoculum. Indeed, polymicrobial inocula have been tested in a range of trials with the aim of exploiting distinct beneficial traits from multiple species [16, 18, 34, 38, 39].

We previously identified phosphate-solubilizing and phosphate-non-solubilizing bacteria that were associated with our study host *Zea mays* [36, 37]. These strains were from genera previously described as containing PGPRs: *Rahnella* [40], *Rhizobium* [41], *Stenotrophomonas* [42] and *Serratia* [43]. In this study they were used to inoculate *Z. mays* seeds in a greenhouse mesocosm experiment and controlled microcosm experiments. We hypothesized that P-solubilizing bacteria could promote plant growth more effectively in orthophosphate-deficient, insoluble P-rich condition when compared to conditions with non-limiting orthophosphate. We also hypothesized that microbial competition could limit the benefits derived from inoculation with PGPRs. We manipulated levels of competition by using varying numbers of isolates in seed inoculants, but we also repeated experiments in sterilized soil and with gnotobiotic plants in microcosms using sterile plant growth media. Microcosm experiments also tested whether competition between inocula affected colonization of plants and whether growth promotion was linked to bacterial density on roots.

## Methods

### Study and bacterial isolates

*Zea mays* var*.* Minipop was used in this study. Bacterial strains had previously been isolated from the roots of *Z. mays* as part of a previous study and were stored as glycerol stocks at −80 °C [36, 37]. Isolates were selected based on previously assayed P-solubilizing phenotypes [36, 37]. Two *Zea* root*-*associated bacterial isolates were selected for high P-solubilizing (P+) phenotype (isolates W and X) and two were selected for the absence of a P-solubilizing (P−) phenotype (isolates Y and Z). These isolates were used singly, and in order to manipulate levels of competition between inocula, were also used in pairwise, three-way and four-way combination treatments.

Isolates were identified to genus level using partial amplification of the 16S rRNA gene. In brief, we conducted colony PCR using primers 27F and 1492R using the conditions described in Marchesi *et al*. [44] (Table 1). PCR products were sequenced by Eurofins Genomics (Ebersbug, Germany). Chromatograms were visualized and trimmed in 4Peaks (https://nucleobytes.com/4peaks/) and forward and reverse sequences aligned using Clustal Omega (https://www.ebi.ac.uk/Tools/msa/clustalo/). Consensus sequences were subject to nucleotide blast searches [45] to search for homologous bacterial sequences.

### Seed sterilization and inoculation

Seeds were aliquoted into batches of 100 and sterilized using bleach and ethanol as described previously [37]. Specifically, each aliquot was submerged in 45 ml of 5 % (free chlorine) household bleach and vortexed for 30 s, before being inverted continuously by hand for 10 min. The bleach was then drained and the seeds were rinsed with sterile water before being resubmerged in 70 % ethanol. Aliquots were then vortexed again for 30 s and inverted for 5 min by hand. The ethanol was drained, and the seeds were immersed in sterile water for 30 s before draining, three times. A subsample of sterilized seeds (*n*=10) were plated onto 2 % LB agar and kept for 2 weeks to validate sterilization; no colonies were produced on these plates. The seeds were then immersed in sterile water and left to imbibe for 48 h in the dark at 10 °C with periodic shaking to aerate. The water was then decanted and seeds were left in the dark at 10 °C for a further 48 h. Seeds were then transferred to either a soluble P or insoluble P modified Murashige and Skoog (MS) medium for germination and screening of contaminants [46]. MS media were composed of a 1 % agar with standard nutrients except that in the insoluble P medium 1.25 mM l^−1^ Ca_3_(PO_4_)_2_ was substituted for the 1.25 mM l^−1^ KH_2_PO_4_ used in the soluble P medium. Plates containing 10–15 seeds per plate were incubated at 28 °C for 72 h in the dark before being transferred to experimental treatments. Only seeds germinated in insoluble P media were used in insoluble P treatments and vice versa.

Stocks of rhizobacteria were refreshed on 2 % LB agar plates. Single colonies were selected and grown for 16 h in 5 ml of 2 % LB at 30 °C and constant 150 r.p.m. shaking. This overnight culture was diluted 1 : 25 in sterile saline (0.85 % w/v NaCl) and 25 ml of this diluted stock was added to 75 ml of sterile water to make the final inoculum. Colony-forming units (c.f.u.) in diluted inocula (0.1 and 0.01 %) were enumerated by plating on LB at 30 °C. In the *Zea* soil mesocosm experiments, inoculation occurred via a soil drench method 4 and 14 days post-sowing for non-sterilized soils and sterilized soils, respectively. One millilitre of the prepared overnight culture was added to 100 ml of 2 % LB and incubated again for 16 h at 150 r.p.m. shaking; 30 ml of the culture was then aliquoted for each litre of inoculant needed in a 50 ml Falcon tube. In the case of mixed treatments, equal volumes of inoculant from each isolate were mixed to make up 30 ml. The overnights were added to a litre of sterile water and shaken thoroughly, prior to being applied to seedlings. Seedlings were distributed equally between trays according to days since emergence. Then 50 ml of inoculant was added to each pot near the base of the seedling and the densities of culturable bacteria were confirmed by dilution plating at inoculation.

### Mesocosm experiment: soil preparation

Four different soil treatments were used in the glasshouse mesocosm part of this study: sterilized or non-sterilized, with either soluble or insoluble forms of P. Ongar Loam was used for the non-sterile treatments, a low-nutrient soil type produced by Binder (Ongar, UK) for dressing cricket pitches (see Table S1 for specifications of Ongar Loam, available in the online version of this article). The sterile soil treatment used the same Ongar Loam that had been steam-sterilized by the supplier. Soils were treated with either the organic phosphate treatment through the addition of Ca(H_2_PO_4_)_2_ (85% purity) or the inorganic treatment of Ca_3_(PO_4_)_2_. Concentrations of each nutrient were calculated by converting 265 kgNHa^−1^, 90 kgKHa^−1^ and 135 kgPHa^−1^ into 0.441 gN kg^−1^ (soil), 0.150 gK kg^−1^ (soil) and 0.225 Pg kg^−1^ (soil), respectively, under the assumption of 0.6 kg of soil being used in each pot. In order to ensure plant release from N and K limitation, soil was added 50 kg at a time into a compost tumbler with 104 g of (NH_4_)_2_SO_4_ and 14.2 g of KCl in a 500 ml solution spread finely on top. For soluble P treatments, 38.8 g of Ca(H_2_PO_4_)_2_ (85 % purity) in a 500 ml solution was also added. For insoluble P treatments 45.4 g of Ca_3_(PO_4_)_2_ (98 % purity) was sprinkled in a powder form over the soil. After mixing (2 min) and decrumbing, soil was potted into 3″×3″×5′ pots.

### Seed sowing

Pots were arranged randomly on trays in a glasshouse. Within each tray soil sterility and inoculation treatment were the same to avoid cross-contamination; however, the position of the pots within the trays was fully randomized (soluble P vs insoluble P), with *n*=10 per soil type (sterility and P solubility) and bacterial treatment. The pots were initially watered from below until wet through. A handful of bleached and sterile water-rinsed sharp sand was placed evenly over the surface of the pot before a seed was sown on top and then covered by the same sterilized sand.

### Glasshouse co-culture regime

The glasshouse simulated 16 h light cycles from 06 : 00 to 22 : 00 with a temperature of 25 °C during the day and 20 °C at night; 400W bulbs were suspended 1.5 m above the growing plants at intervals of 1.5 m. The lights turned off if ambient light levels exceeded 450 lux. An hour of dawn warm-up and dusk cool-down between 06 : 00 and 07 : 00, and 21 : 00 and 22 : 00 respectively were used. Water was applied to the tray below the pots each day, with approximately 100 ml per pot. Tray position was shuffled randomly every 7–10 days.

### Data collection

*Z. mays* plants were left to grow for 28–29 days as we wanted to focus on vegetative growth to enable simple comparisons based on shoot mass and to ensure that P did not become limiting in all treatments. Individuals were washed and dried with a towel before being bisected into root and shoot. Fresh total mass, shoot mass and root mass were measured. Individuals were placed into separate sheets of newspaper and were left to dry for 7–8 days in a 70 °C oven. Dry total mass, root mass and shoot mass were then taken for each individual.

### Microcosm experiment

The same *Z. mays* variety was used as the mesocosm experiment. The same four bacterial isolates were also used in isolation. As above, we manipulated opportunities for competition by including all pairwise, three-way and four-strain combinations as seed inocula. Treatments were set up with least 16 replicates, with the exception of the uninoculated controls, which used 32 replicates.

Seeds were sterilized and incubated as described above before being individually grown on 9 cm agar plates containing either soluble P or insoluble P MS media using Oxoid bacteriological agar (Oxoid, Basingstoke, UK). A soldering iron was used to create a notch (approximately 5 mm wide and deep) in both the lid and tray of each plate, resulting in a hole in the side of the plate when closed [47]. Seedlings were taken from the incubator and positioned with their shoot emerging from the soldered hole and their root in contact with the medium in individual plates. The plates were stacked in groups of 10 and wrapped in Parafilm, with aluminium foil wrapped around each stack to prevent light reaching the roots, before being stacked on their sides in a positive-pressure and high-humidity Perspex box for 24 h (Fig. S1).

Inoculants were prepared from the same regenerated glycerol stocks on agar as used in the mesocosm experiment. The same 4 colonies were used to inoculate a 24-well plate, with 1 ml of 2 % LB in each well. After 16 h at 30 °C and 150 r.p.m. shaking, media were diluted 1 in 20 in sterile saline and diluents pipetted along the length of the roots.

Seedlings were kept at 24 °C with 12 h day cycles. Dawn and dusk were simulated with a gradual increase or decrease of light for 30 min at the start and end of each day cycle. After 24–26 days of growth, plant root subsamples were washed of all MS media in warm water. Individuals were then bisected, and fresh total, shoot and root masses were taken. Root systems were transferred to 2 ml homogenization tubes containing 750 µl of sterile saline (0.85 % NaCl) and destructively sampled using a beadbeater (Qiagen Tissue Lyzer, Manchester, UK) with 250 µl 0.1 mm diameter ceramic beads (Zymo, Cambridge Bioscience, UK). Root macerates were serially diluted in saline and plated on LB (Lysogeny Broth) agar.

### Statistical analyses

The mesocosm experiment with non-sterilized soil, the mesocosm experiment with sterilized soil and the microcosm experiment were all analysed separately. The non-sterile and sterile mesocosm experiments were not compared directly due to the age difference of plants at inoculation. All statistical tests were carried out within R4.0.3 (R Core Team, 2020). Generalized linear models (GLMs) were used in base statistics to analyse all other wet and dry mass against P solubility and bacterial treatments. We examined the variation in investment between shoots and root ratios by testing how bacterial inocula and P solubility affected the relationship between dry shoot mass and dry root mass, i.e. using dry root mass as a covariate to explain shoot mass. This avoids statistical issues of analysing ratios directly (e.g. lack of normality). Post-hoc treatment contrasts were used to assess effect sizes and to compare differences between uninoculated controls and inoculation treatments.

In the microcosm experiment, plant growth data were analysed as wet mass only, using the methods above, as it was not possible to both dry plants and fully recover endophytic bacteria from roots. Bacterial densities tended not to be normally distributed in the microcosm experiment and were strongly bimodal. We used χ^2^ tests to examine patterns in co-colonization among different bacteria inoculate across the experiment. In order to make comparisons between treatments in colonization efficiency we used GLMs with binomial errors to assess proportional colonization, or non-parametric Kruskal–Wallis tests to make comparisons on total bacterial counts. Just under 10 % of plants in the microcosm experiment were colonized by a fungal contaminant (42/537 plants). While fungi did not affect colonization of roots by microbes, they did reduce plant wet mass (GLM: *F_1,535_* =53.8, *P*<0.0001), so these replicates were excluded from any analyses of plant growth. Mean values given in the results section are ±1se.

## Results

### Effects of phosphorous availability and inocula on plant growth in non-sterilized soil

**Fig. 1.**
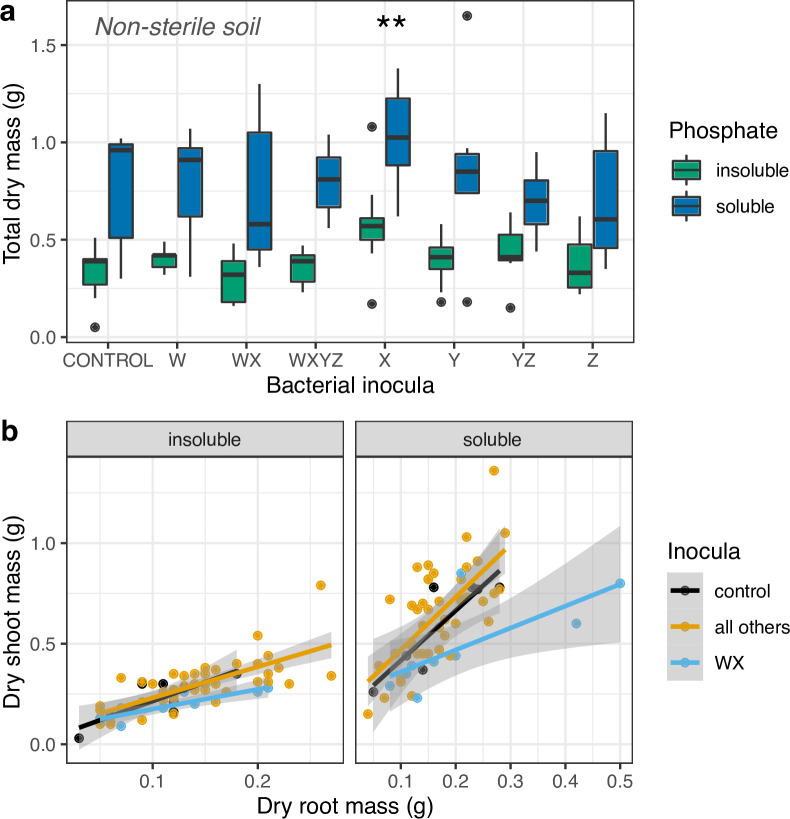
(**a**) A box-and-whisker plot to show maize Minipop dry mass (**g**) on a log_10_ scale as a response to bacterial treatment in the non-sterilized mesocosm experiment. Letter code combinations on the *x*-axis indicate the combination of isolates used to inoculate seeds (see Table 1). Inoculation with *Serratia* isolate X led to plants having significantly greater mass than controls, while plants in soluble P treatments had a significantly greater mass than plants in insoluble P treatments, respectively (*P*<0.001, indicated by **). Individual points denote ‘outliers’ that are either greater than the upper quartile+1.5 ×the interquartile range (IQR) or the lower quartile−1.5×IQR. (**b**) The relationship between dry shoot mass and dry root mass for maize inoculated with different microbes. Inoculation with isolate mix WX (light blue) produced a significantly different relationship between root and shoot mass relative to controls; inoculation with all other isolates and isolate mixtures (in yellow) is shown for comparison. Data are independent plants and lines are fitted linear models from the GLMs with shading showing 95 % confidence intervals.

We tested the effects of P availability and different bacterial inocula on the growth of *Zea* in a mesocosm experiment in non-sterilized soils. Firstly, we confirmed that P was limiting in our insoluble P treatment: the total dry mass of maize plants grown in insoluble P soil was half that of plants grown in soluble P soil (0.38±0.02 g compared with 0.76±0.04 g, *F*_1,121_=88.4, *P*<0.0001; Fig. 1a). Bacterial inoculation also affected dry mass (*F*_7,114_=2.61, *P*<0.05). In contrast to our first hypothesis that P solubilization would be most beneficial when P was limiting, the effect of microbial inoculation was consistent across P treatments (test for interaction between P treatment and bacterial inoculation *F*_7,107_=0.35, *P*=0.93; Fig. 1a). Inoculation with *Serratia* isolate X, which effectively solubilizes phosphate (Table 1), was the only treatment which increased dry weight relative to the control (post-hoc contrast *t*=3.32, *P*<0.01). To illustrate: the mean total dry mass in the isolate X treatment was 0.79 g (±0.08) compared with 0.49 g (±0.08) in the control (Fig. 1a).

Total fresh mass (log-transformed) showed qualitatively similar patterns to plant dry mass. Fresh mass was higher in soil amended with soluble P compared to soil amended with insoluble P (8.59±0.47 g compared with 3.9±0.23 g, *F*_1,121_=94.8, *P*<0.0001). Bacterial inoculation also affected fresh mass (F_7,114_=2.80, *P*<0.05), and this was consistent across both P treatments, i.e*.,* there was no significant interaction with P treatment and bacterial inoculation (*F*_7,107_=0.23, *P*=0.98). The effects of bacterial inoculation on wet mass were similar to the results for dry weight, in that inoculation with *Serratia* isolate X gave the biggest increase to plant growth (post-hoc contrast *t*=3.7, *P*<0.001), although treatment WXYZ also increased final fresh weight (*t*=2.12, *P*<0.05).

In the mesocosm experiment we also explored the relative investment in roots and shoots by testing whether dry root mass (log-transformed) affected dry shoot mass (log-transformed). Phosphorous availability increased relative investment in shoots (*F*_1,113_=117, *P*< 0.0001 Fig. 1b), confirming that root foraging is more efficient when phosphorous is readily available in a soluble form. However, phosphate treatment did not interact with bacterial inoculation (*F*_7,98_=1.70, *P*=0.54) or with root mass (*F*_1,105_=2.95, *P*=0.09). Bacterial inocula affected the relationship between dry shoot and dry root mass (bacteria×root mass interaction, *F*_7,106_=3.51, *P*<0.01; Fig. 1b). However, this was driven by the results for the WX inoculum; pooling all the other inocula treatments did not affect the explanatory power of our statistical models (*F*_10,106_=1.41, *P*=0.19; Fig. 1b). Two data points in the soluble-P treatment had a large influence on this analysis (Fig. 1b). Nevertheless, putting aside the relationship with dry root mass, inocula WX consistently lowered shoot mass across both P treatments suggesting this treatment behaved differently to the others (*F_2,118_* =9.42, *P*<0.0001; Fig. 1b). There was no evidence that any of the other inocula affected the slope of the relationship between shoot and root mass (post-hoc contrast of pooled inocula versus control *t*=0.125, *P*=0.90).

### Effects of phosphorous availability and inocula on plant growth in sterilized soil

**Fig. 2.**
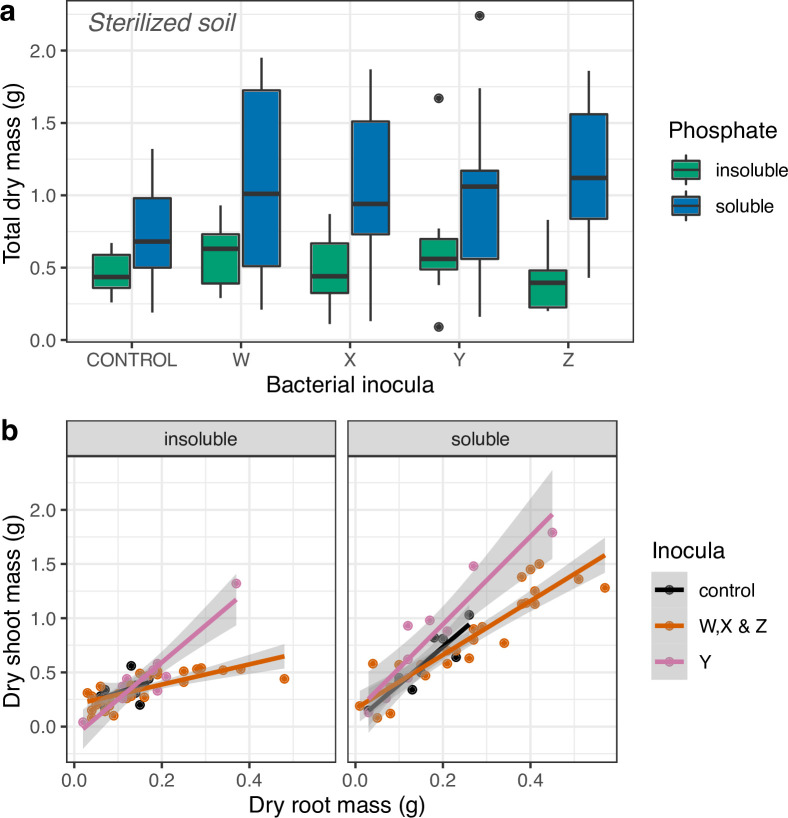
(**a**) A box-and-whisker plot to show maize Minipop dry mass (**g**) as a response to bacterial treatment in the sterilized mesocosm experiment. Bacterial treatments did not significantly affect total dry mass. Soluble P soils produced significantly heavier plants than insoluble P soils (*P*<0.001). Individual points denote ‘outliers’ that are either greater than the upper quartile+1.5×the interquartile range (IQR), or the lower quartile−1.5×IQR. (**b**) The relationship between dry shoot mass and dry root mass for maize inoculated with different microbes. Inoculation with isolate Y (pink) produced a significantly different relationship between root and shoot mass relative to controls; inoculation with isolate W, X or Z (in gold) is shown for comparison. Data are independent plants and lines are fitted linear models from the GLMs with shading showing 95 % confidence intervals.

In the sterilized soil experiment, total dry mass (log-transformed) was again higher in soil amended with soluble P in comparison to soil amended with insoluble P (1.00±0.09 g compared with 0.52±0.04 g, *F*_1,92_=19.0, *P*<0.0001; Fig. 2a), but was not affected by any bacterial treatments compared to the control (*F*_4,88_=0.31, *P*=0.87). Similarly, plant fresh mass was higher in soluble P soil compared with insoluble P soil (10.6±0.96 g and 5.1±0.40 g respectively, *F*_1,92_=19.7, *P*<0.0001). Fresh mass did not vary with bacterial inoculation treatment (*F*_4,88_=0.43, *P*=0.78).

Relative investment in shoots and roots was also affected by P solubility in sterilized soils, with dry shoot mass (log-transformed) predicted by an interaction between dry root mass and P solubility (P treatment×root mass interaction *F*_1,82_ = 31.6, *P*<0.001; Fig. 2a). Overall investment in shoot mass increased more quickly with increasing root mass in soluble P soils (i.e. affected the slope of the relationship between the two). Thus, larger plants could invest more in shoots, as might be expected if fewer roots are needed to harvest readily available P (Fig. 2b). This is a subtly different result to the non-sterile soil experiment in which P solubility increased the overall investment in shoots relative to roots.

Bacterial inocula also affected the relative investment in roots and shoots in sterilized soil mesocosms (bacteria×root mass interaction *F*_1,83_ = 8.60, *P*< 0.001; Fig. 2b). Inspection of data and post-hoc contrasts suggested that isolates W, X and Z showed similar patterns to each other. This was confirmed through a test that examined the effect of pooling these treatments on the explanatory power of our GLM (*F*_8,74_ = 0.01, *P*=0.97; Fig. 2b). *Rhizobium* isolate Y, on the other hand, produced greater masses of shoots relative to mass of root in both P treatments (*F*_2,87_ = 16.5, *P<*0.001; Fig. 2b), suggesting a beneficial impact that allowed increased relative production of shoots in sterile soil.

### Effects of phosphate availability and inocula on plant growth in a microcosm experiment

**Fig. 3.**
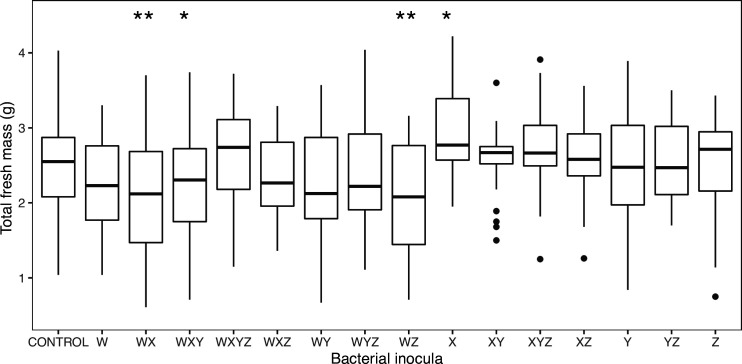
A box-and-whisker plot showing variation in effect of bacterial inoculants on maize Minipop wet mass in the microcosm experiment. Significance of post-hoc treatment contrast relative to the control is shown above boxes (*P<0.05, **P<0.01). Individual points denote ‘outliers’ that are either greater than the upper quartile+1.5×the interquartile range (IQR) or the lower quartile−1.5×IQR. Phosphate treatments were pooled in this analysis, as they had no detectable effect on plant growth in this experiment.

In the microcosms, plants were grown in sterile artificial media in a controlled temperature room, rather than in pots in a greenhouse, in order to test whether competitors affected plant colonization in controlled conditions, and to test whether plant growth promotion was dependent on robust colonization of root material. Note that plant growth was recorded as wet mass in these experiments, as roots were destructively sampled in order to quantify bacterial colonization. Phosphate solubility was not a predictor of any response variables, including final fresh mass (*F*_1,452_=2.02, *P*=0.16) and phosphate treatments were therefore pooled in all subsequent analyses. Nevertheless, the various bacterial inoculants did have an impact on fresh mass (*F*_15,453_=3.19, *P*<0.0001; Fig. 3). We recovered one of the patterns seen in the mesocosm experiments in that *Serratia* isolate X also increased final fresh mass of *Zea* (Fig. 3, *Serratia* inoculated plants – mean 2.89 g; controls 2.53 g). However, a number of treatments inhibited the growth of plants relative to controls – specifically bacterial treatments WX, WXY and WZ (Fig. 3). All inoculant mixtures containing *Rahnella* isolate W (bar the four-isolate mixture) were parasitic in the sense that they reduced final mass relative to controls (estimate of difference −0.3 g, *t=*−2.96*, P*<0.01). Fresh shoot : root mass ratios were not different between inoculation treatments (*F*_15,445_=1.1, *P*=0.35, respectively).

### Interactions between microbes in microcosms

**Fig. 4.**
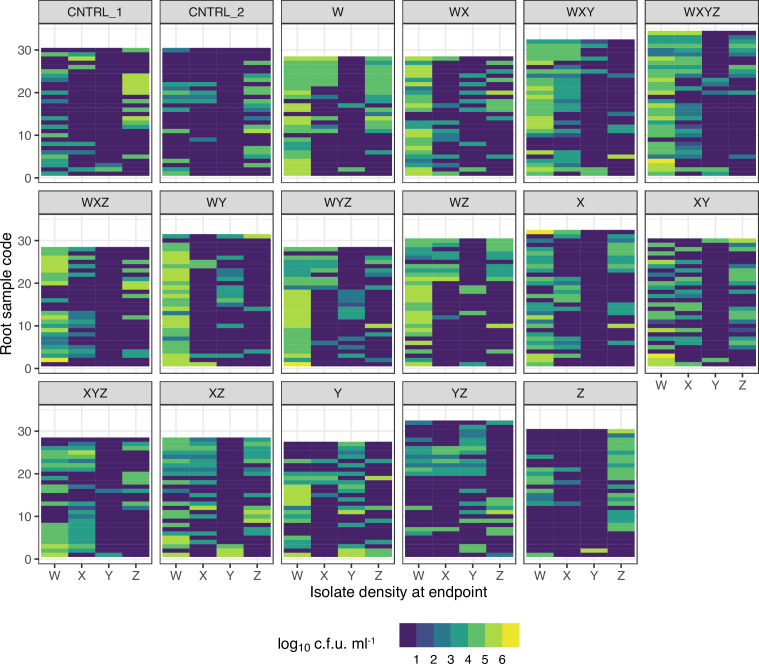
Heatmap of plant colonization efficiency by four focal rhizobacteria isolates in a gnotobiotic microcosm experiment using artificial Murashige and Skoog agar and maize Minipop seedlings. Makeup of the initial inoculum is indicated with the letter codes in the banner of each sub-panel. Microbial densities are in log_10_ c.f.u. ml^−1^ of root homogenate.

Inspection of colony count data in these experiments shows that experimental inoculations were key to determining which isolates colonized plants (Fig. 4). Nevertheless, there was clearly secondary movement of inoculated microbes between plants post-inoculation, so that plants originally inoculated with isolate X, for example, were eventually colonized by isolate W (Figs 4 and S2). Despite this, we predominantly recovered the morphotypes used to inoculate experimental plants, suggesting that the positive pressure in our experimental set-up largely prevented colonization by other environmental microbes (Fig. S1). In addition, semi-natural colonization post-inoculation also proved to be very instructive in terms of revealing ecological interactions between isolates, such as competition or facilitation (Table 2).

**Table 2.** A table showing associations between rhizobacterial isolates in the microcosm community experiment. Isolate associations are based on observed co-colonization of independent root samples, not treatment co-inoculation; *P* values for χ^2^ tests are corrected for multiple tests using Benjamini and Hochberg’s method [66]

Isolate pair	Direction of association	χ^2^ value	GLM – effect size (se) with *t*-test
**X+Y**	Negative	17.3 ***	−0.11 (0.03)**
**X+W**	Positive	41.6 ***	0.28 (0.04)***
**X+Z**	–	0.6	0.07 (0.04)
**W+Y**	–	1.16	0.08 (0.03)*
**W+Z**	–	0.23	0.003 (0.05)
**Y+Z**	Negative	23.4 ***	−0.01 (0.03)**

There was evidence that competition between isolates could prevent root colonization. For instance, *Stenotrophomonas* isolate Z appeared to have a ruderal ecology and more effectively colonized plant roots that were not co-inoculated with other strains. This isolate was also the most effective secondary colonizer of control plants (Figs 4 and S2). The density of this strain increased as the number of isolates used within each seed inoculum decreased (Kruskal–Wallis test χ^2^=21.2, df=4, *P*<0.0001; Fig. S3). Note that the presence of isolates W and X on roots at the end of the experiment did not affect the colonization patterns of isolate Z, it was the inoculation treatment that was important (see tests in Table 2, Fig. 4). In addition, the distribution of *Rhizobium* isolate Y indicated that it could be competitively excluded by isolates X and Z (Table 2), since *Rhizobium* Y was found where X and Z isolates were absent more than would be expected by chance (see tests in Table 2, Figs 4 and S2 boxplot). In contrast to Z, isolate Y was a poor colonizer and had very low densities outside of ‘Y only’ and YZ treatments (Figs 4 and S2).

**Fig. 5.**
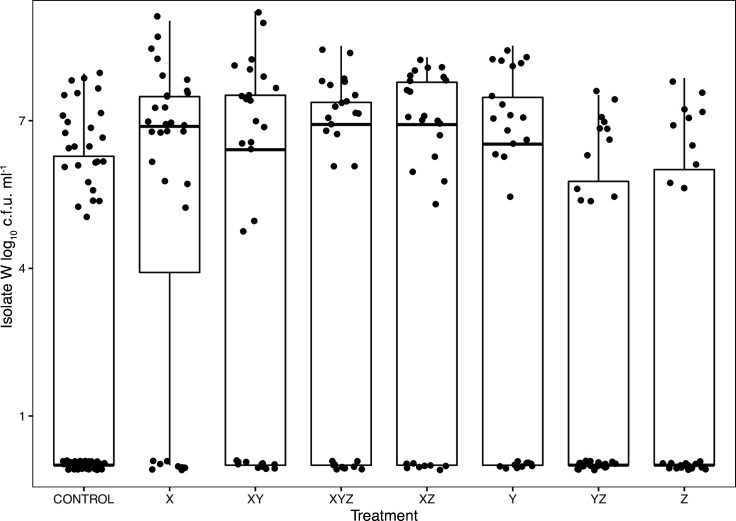
Boxplot showing secondary colonization of maize roots in microcosms by isolate W; boxplots are overlaid with jittered raw data. These data derive from treatments in which isolate W was not applied as a seed inoculant and so all colonization is between plants during the experiment. Treatment letters on the *x*-axis refer to bacterial isolates used in initial inocula. There was a significant difference in secondary colonization across treatments (Kruskal–Wallis test χ^2^=34.3, df=7, *P*<0.0001).

Although a key aim of this study was to test the importance of between-species competition, we also found evidence that bacterial interactions were not solely or even predominantly competitive on plants. For instance, *Rahnella* isolate W had positive associations with both isolates X and Y: W+X and W+Y co-colonization occurred more often than would be expected by chance (Table 2, Fig. S2). *Rahnella* W was a very good secondary colonist of plants that were not initially inoculated with this isolate. Secondary colonization patterns also showed evidence of facilitation by *Rahnella* W by isolates X and Y: inoculation treatments containing X or Y had more secondary colonization by *Rahnella* W relative to other treatments (Fig. 5, Kruskal–Wallis test χ^2^=34.3, df=7, *P*<0.0001). Inoculation treatments X, XY, XYZ and Y were frequently colonized with high densities of isolate W (median densities in the region of 10^7^ c.f.u. ml^−1^). Control plants, and those inoculated with Z or YZ isolates, also had a lower proportion of plants colonized by isolate W (GLM with quasibinomial errors, *F*_1,6_=39.2, *P*<0.001).

### Effect of secondary microbial colonization on plant mass

**Fig. 6.**
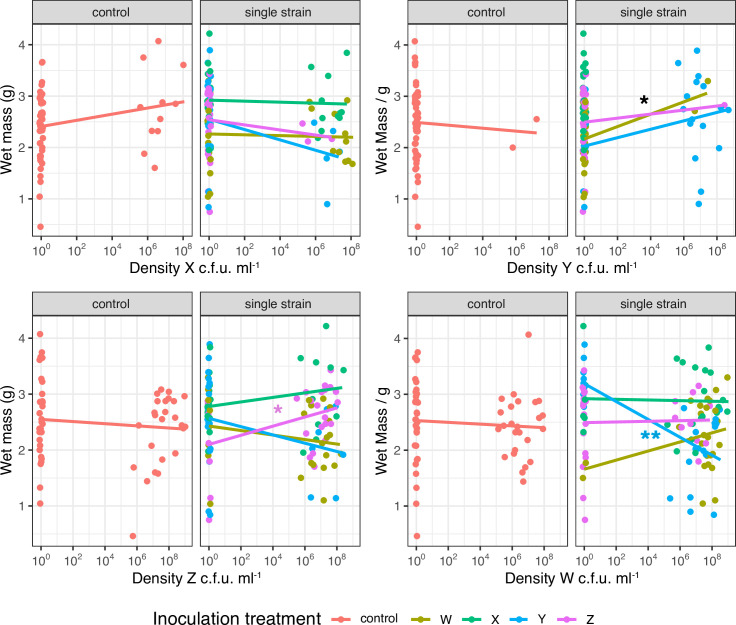
Effect of plant colonization by microbial inoculants on growth of maize Minipop in microcosms. Data are from treatments inoculated with single isolates only, density is log_10_ colony-forming units (c.f.u.) ml^−1^ homogenate. Asterisks indicate significant associations between bacterial density and plant wet mass *(*i.e*.* significant non-zero slopes of fitted lines). Colour coding indicates which inoculation treatments we saw an effect of bacterial density in, i.e*.* black *P<0.05 for all inoculation treatments (density Y); purple *P<0.05 inoculation treatment Z (density Z); blue **P<0.01 inoculation treatment Y (density W).

While we saw that seed inoculation treatment did have some effect on plant wet mass in the analysis above, there was no evidence that the densities of our mutualist PGPR *Serratia* isolate X on roots were correlated with plant mass (*F*_1,494_=0.002, *P*=0.96). While the interactions between colonization, inoculation and plant growth were too complex to unpick statistically in the experiment as a whole, we did see patterns in the simpler subset of treatments that used single isolate inocula (Fig. 6). Colonization by *Serratia* X (as opposed to inoculation) did not clearly increase plant growth, but there was a trend in that direction for the uninoculated control plants (effect size 0.06, *t*=1.76, *P*=0.06; Fig. 6), which suggests that our observed estimate of the effect of X inoculation on maize growth is conservative. Colonization by *Rhizobium* isolate Y improved plant growth in all single isolate treatments (*F*_1,153_=4.83, *P*=0.029), while there was evidence that colonization by Z increased growth in treatment Z only (effect size 0.10, *t*=2.31, *P*=0.02; Fig. 6). In contrast, colonization by W was parasitic in terms of reducing plant growth in treatment Y only (effect size −0.13, *t*=−2.83, *P*=0.006; Fig. 6).

The parasitic *Rahnella* W tended to block any positive effects of other isolates on plant growth. This means that we can better see how colonization with isolate X affected plant growth if we exclude plants originally inoculated with isolate W. In this subset of replicates, all plants inoculated with *Serratia* X (alone or in a combination) had increased growth relative to controls (inoculation treatment effect *F*_2,242_=3.42, *P*=0.034; effect of *Serratia* X relative to controls 0.34, *t*=2.71, *P*=0.0073), as we saw in the whole experiment (Fig. 3). In contrast, high *in planta* densities of *Serratia* X were not typically associated with increased plant growth. Densities of *Serratia* X were positively associated with plant growth in uninoculated controls but did not affect growth in inoculation treatments containing X, and were negatively associated with plant growth in treatments Y, YX and Z (treatment×density interaction *F*_2,239_=4.17, *P*=0.017; Fig. S4). Overall, then, *Serratia* X did not require high densities to boost plant growth, but exposure of seeds or uninoculated plants to this strain could have lasting effects in this experiment.

## Discussion

Phosphate solubilizing ability was not a reliable predictor of which rhizobacterial isolates could stimulate plant growth in this study. While the only plant growth promoter we identified in this study could efficiently solubilize phosphorous, its ability to increase plant growth did not depend on the availability of soluble phosphorous and nor did it increase plant growth only when P was limiting. In fact, the presence of potentially competing root inocula, and the physical/microbiological qualities of soil (steam sterilized or not) were more important in determining whether this isolate could increase plant growth or not. PGP effects are notoriously hard to replicate in different conditions [28], but nevertheless *Serratia* isolate X increased growth in two independent experiments, irrespective of whether P was limiting or not limiting.

These results indicate that the plant growth promotion of *Serratia* isolate X was unrelated to its ability to solubilize P. When soluble P was added in quantities that are generally accepted to completely remove any P deficiencies [48], this isolate could still increase plant growth via other means (e.g. siderophore production, antifungal activity or biostimulant [21]). Notably, phosphate solubilization ability is routinely used as a means of identifying PGPR [49, 50]. Different studies have very different conclusions on whether phosphate-solubilizing bacteria promote growth via phosphate solubilization. Recent studies have found that the application P-solubilizing bacteria can increase increased yield regardless of the phosphate source in the soil [34, 40]. Similarly, *Bacillus* spp. may or may not increase P uptake, depending on the study context [49, 51], while a number of studies show that the benefits of PGPR increase in fertile soils, i.e*.* when nutrients are less limiting [33, 52, 53]. Indeed, our study suggests that using P solubilization as a means of shortlisting potential plant mutualists must be treated with caution, as P solubilization may not be the basis of all mutualistic interactions with plants. However, using high P solubilization to shortlist strains might be a convenient morphological route to identify useful isolates from particular microbial families. For example, the *Pseudomonadaceae* and *Enterobacteriaceae* tend to be efficient at solubilizing phosphorous [36] and are widely exploited in PGPR. There are a wide range of mechanisms linked to phylogeny that could explain why the practice of isolating phosphate-solubilizing bacteria has been successful in the past [36].

In contrast to the unimportance of P limitation, interactions between inoculating strains consistently affected plant growth and growth promotion by *Serratia *. In this study mixed inocula were used as a means of manipulating levels of competition on plant roots, but these results are also relevant to the applied practice of using mixed inocula as a means of providing more robust plant growth stimulation. Previous studies have cautioned against the use of mixtures in plant inocula, arguing that single strain inocula provide more robust stimulation of plant defences against insects [54]. Interactions may depend on the genetic distance between organisms combined in inocula. For example, combinations of bacteria and fungi have been successful in promoting plant growth [16, 55]; combinations of either multiple *Pseudomonas* or multiple *Bacillus* isolates have proven to be more potent in terms of suppressing plant diseases than single isolates, but combinations of Gram-negative and Gram-positive microbes are often not optimal [18, 39, 56]. Finally, a recent meta-analysis suggest that combinations of P-solubilizing and nitrogen-fixing microbes are particularly potent for growth promotion [53]. The practice of screening isolates for competitive interactions *in vitro* before combining them in mixed inocula therefore has much to recommend it [40].

A key difference between other studies and our own is that we used combinations of uncharacterized rhizobacteria to test whether competition could affect colonization of rhizobacteria and growth promotion, whereas applied studies tend to use combinations of strains with known mutualistic potential. This study provided clear evidence that multiple rhizobacterial isolates were affected by other microbes: one preferred uncolonized roots and another showed patterns of antagonistic competition, while three out of four isolates were engaged in positive or mutualistic interactions. This matches field studies of crop plants that show that co-association patterns are an important feature of rhizobacterial communities [57]. A surprising but robust result of this study was that mutualistic interactions between bacterial isolates proved more durable and important than mutualistic interactions between one focal PGPR isolate and the host plant. On reflection, this makes ecological sense; interactions between generalist bacteria in the rhizosphere are likely to be more important for immediate growth and fitness of microbes than any long-term and diffuse fitness benefits arising from stimulating host plant growth. This is because selection favouring mutualistic interactions between microbes and hosts can only operate consistently in the long term when there is reliable vertical transmission of microbes [58]. Vertical transmission provides the key link between host and symbiont fitness, provided that greater plant biomass increases the population size of associated symbionts [58]. While vertical transmission is quite widespread for mutualistic fungal endophytes [59, 60], obligate vertical transmission is believed to be rare in plant-associated bacteria [61]. Plants predominantly acquire bacteria via horizontal transmission [61], so associations with hosts are fluid and transient in evolutionary time, making short-term interactions with other microbes more significant. Agricultural inocula are unlikely to contain parasitic strains, so facilitation of harmful microbes is unlikely to occur among commercial inoculants. Nevertheless, all the strains in this study were recovered from maize roots, so these kinds of interactions between naturally occurring harmful isolates and PGPR are plausible in the field and could in part explain the variable efficacy of PGPR.

The use of soil-based greenhouse experiments and *in vitro* plant microcosms in this study provided complementary data that helped unpick the ecological basis of variable plant growth stimulation. Consistent plant–microbe interactions were seen with both approaches. For example, the beneficial impact of isolate X on plant growth was consistently masked by co-inoculation with the more parasitic isolate W. Facilitation of a parasitic W by PGPR X explains the results seen in soil experiments, as well as more robust parasitism in microcosms. In addition, competitive interactions between microbes could also limit the effects of bacterial inoculation on plant growth, as seen in other studies [31]. For example, *Rhizobium* Y was the only isolate that showed a positive correlation between colonization and plant growth in microcosms. Nevertheless, the microcosm experiments showed that this isolate was susceptible to antagonistic competition from other bacteria. The hypothesis that plant benefits from this strain are limited by competition is supported by the experiment with sterilized soil, which, despite the shortcomings of this method in term of altered soil chemistry [62], showed that *Rhizobium* Y improved the shoot : root mass ratios relative to controls and other isolates when competitors are suppressed. One result that was not duplicated in soil experiments was the parasitic effect of *Rahnella* W on the plant growth seen in microcosms. Nevertheless, this strain could be largely commensal in the glasshouse due to the functional complexity of microbial communities or the increased availability of carbon in the glasshouse experiment under natural light, and could be opportunistically pathogenic in certain conditions. A possible drawback of the microcosm experiment was that we did see some secondary colonization of plants by our focal species during experiments. Since we recovered our experimental strains, this indicates that movement of microbes occurred within the outer containers that hosted Petri dishes under positive pressure (Fig. S1). While this means that the colonization of plants by inoculants was not fully controlled, this movement of microbes mimics the movement of microbes that occurs to a much large extent in all pot and field experiments using PGPR. This secondary colonization revealed a great deal about the ecology and interactions of the inoculating microbes. Importantly, the effects of initial seed inoculation were robust and detectable despite this movement, indicating that this experiment does provide valuable data.

One factor that we not able to assess in these short-term glasshouse and laboratory experiments is the effect of bacterial phosphate solubilization on long-term nutrient availability. Microbial turnover can increase the availability of P in long-term experiments, as shown in a 13 year study of temperate grassland, which compared sterilized and non-sterilized soils and measured orthophosphate levels throughout time [63]. Without a healthy microbial community, orthophosphate eventually dropped below detectable levels in the sterilized treatment, whereas non-sterilized soils maintained a relatively constant amount of P [63]. Estimates put bacterial P as making up 2–10 % of total soil phosphorous, although it has also been shown that this level can rise to as much as 50 % under certain circumstances [64]. In contrast, earlier studies suggested that the microbial community competes directly with the plant for orthophosphate, thus inhibiting rather than stimulating plant growth in orthophosphate-deficient soils [65]. This study conducted experiments with actively growing plants in pots, with an expanding microbial population, so potentially any phosphate solubilized by rhizobacteria is retained in microbial biomass and might not be released until roots senesce. It remains to be seen whether inoculation of crops with PGPR that can solubilize phosphate actually increases phosphate ability in the field in the long term.

### Conclusion

Here we tested whether phosphate-solubilizing rhizobacteria provide benefits to plant growth, depending upon levels of bacterial competition and availability of soluble phosphate. We could not support the hypothesis that P solubilization provides benefits to plants in the short term and suggest that this trait may simply be a way of identifying rhizobacteria isolates from families likely to contain mutualists (* Enterobacteriaceae *, *Pseudomonadaceae *). Additionally, positive interactions between microbes (facilitation of parasitic strains) are at least as important as competition in terms of limiting plant growth stimulation by specific seed inoculants. Finally, an ecological assessment of rhizobacterial interactions can be cheaply and effectively carried out in high-throughput microcosm experiments. Microcosm experiments can indicate whether isolates are sensitive or robust in the face of competition, how effective they are at colonizing plants and how they are likely to interact with focal plant parasites.

## Supplementary Data

Supplementary material 1
