# Supplementary material for: Competition and co-association, but not phosphorous availability, shape the benefits of phosphate-solubilizing root bacteria for maize (Zea mays)
Source: Access Microbiol. 2023 Dec 8;5(12):000543.v3. doi: 10.1099/acmi.0.000543.v3 (PMC10765048; doi:10.1099/acmi.0.000543.v3)

Supplementary Material

Supplementary Table 1. A table showing the soil specifications of non-sterilised Ongar Loam™

Determinand	Unit	Quantity
**Copper**	mg/kg	26
**Nickel**	mg/kg	13
**Zinc**	mg/kg	91
**Magnesium**	mg/l	250
**Phosphorous**	mg/l	88
**Potassium**	mg/l	1100
**Nitrogen**	mg/kg	3890
**Organic Matter**	%	7.9
**pH**	-	7.8
**Sand Content**	%	47
**Silt Content**	%	27
**Clay Content**	%	26
**Retained on 2mm**	%	5.7
**Retained on 20mm**	%	<0.1
**Retained on 50**	%	<0.1

Supplementary Figure S1

Photos to show (a) an example of a box in the microcosm experiment and (b) how the boxes sit within the controlled environment room with pumps maintaining positive pressure.

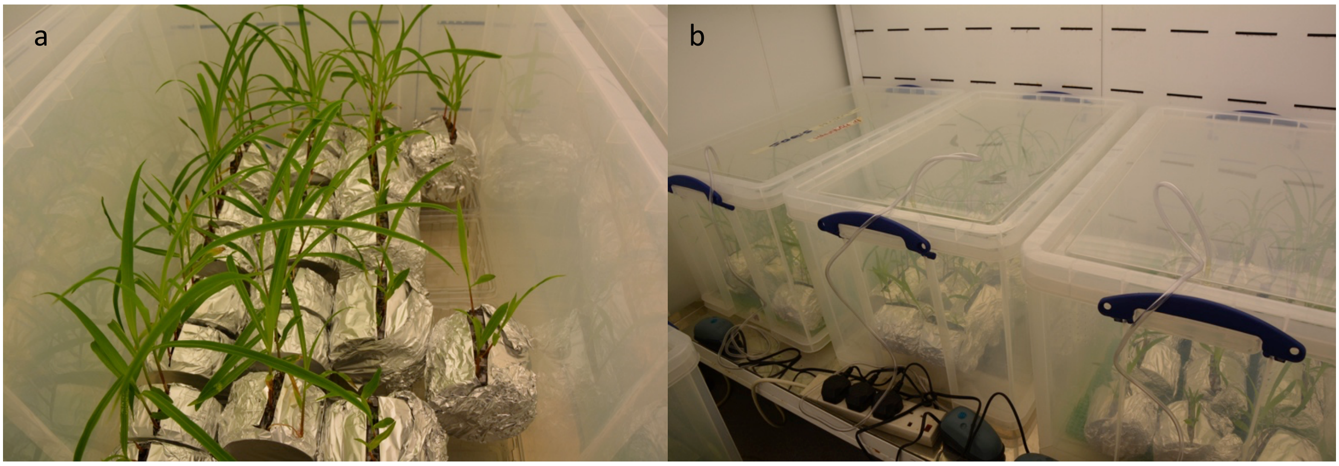

Figure S2. Boxplots of bacterial density of isolates W, Z, Y and X on maize roots and how this varies with seed inoculation treatment.

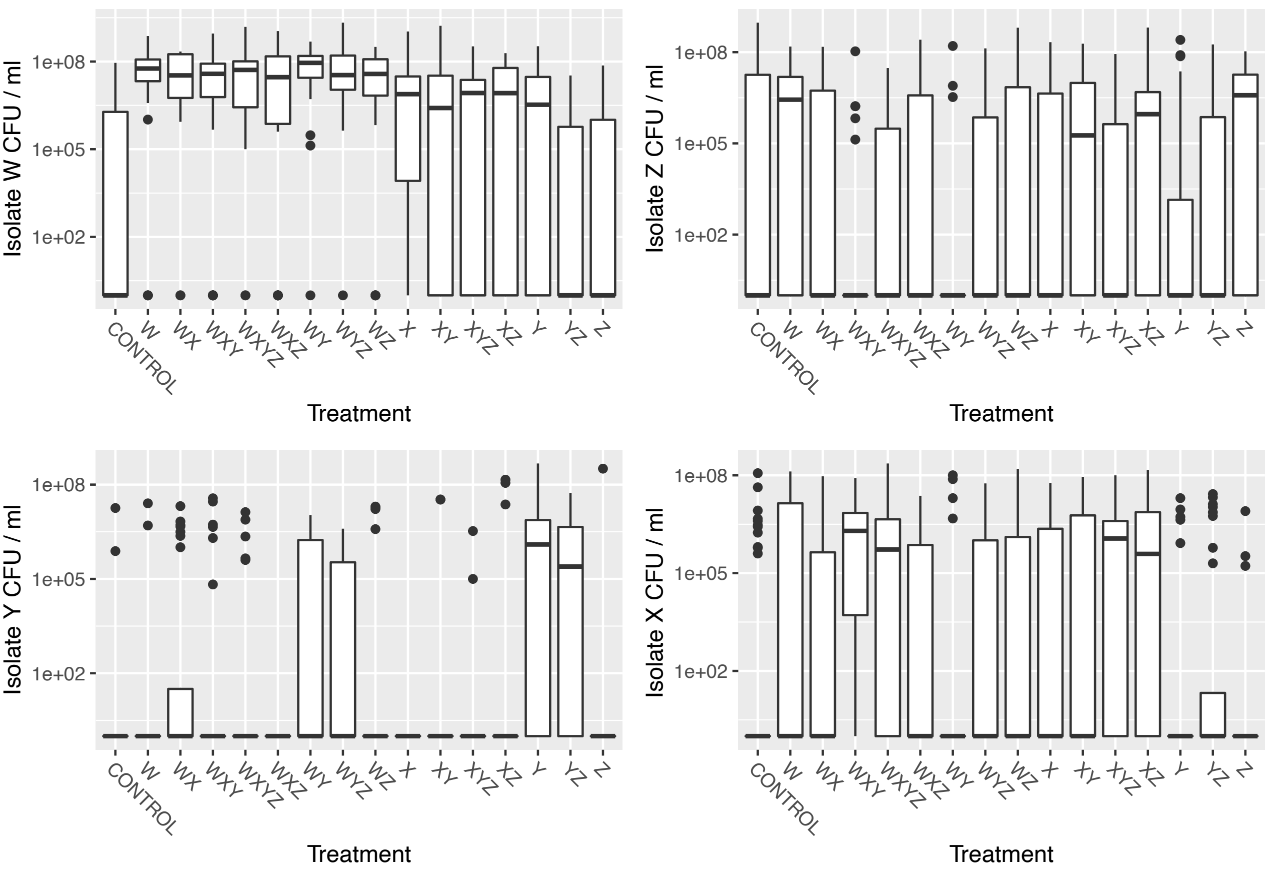

Figure S3. A violin plot of bacterial density of isolate Z on maize roots and how it varies with number of possible competitors during seed inoculation. Violin plots show the probability density of the bacterial counts, which are also shown as jittered circular symbols. The black square symbols correspond to the right-hand y axis and show the proportion of plant roots with non-zero counts of isolate Z. Note that control seeds were not inoculated with any experimental isolates, colonization occurred semi-naturally within experimental chambers.

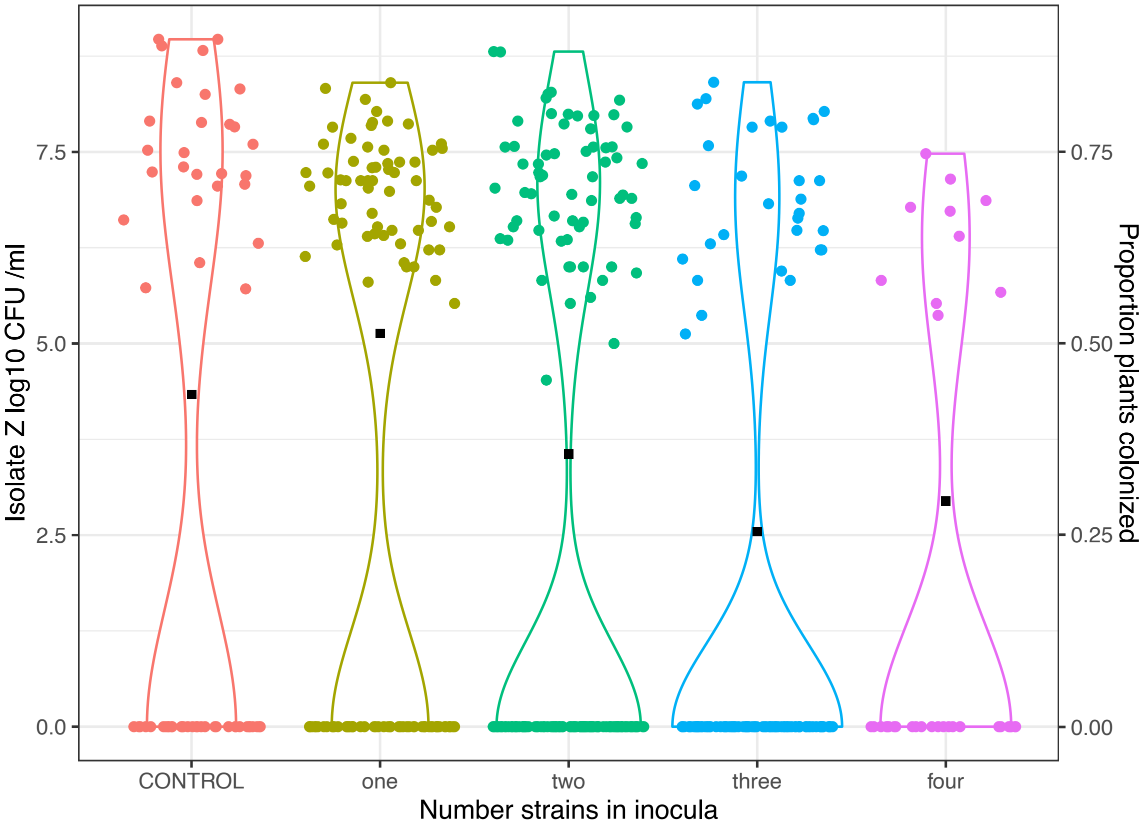

Figure S4. Relationship between density of the potential PGPR isolate X on roots and mass of maize Minipop seedlings in experimental microcosms. Treatments refer to exposure to bacterial inocula at sowing. All treatments inoculated with the parasitic isolate W have been excluded from this analysis. Panels refer to uninoculated controls, all treatments inoculated with X (=”someX”) and treatments inoculated with Y or Z (but not W or X). Lines are fitted linear models.

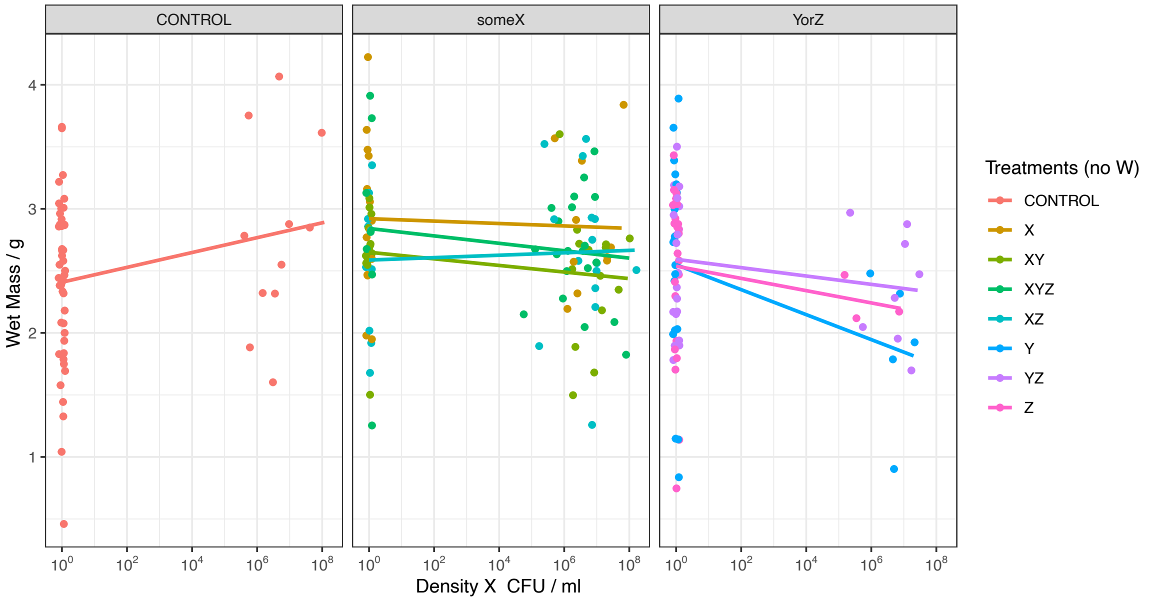

Supplement: Supplementary material 1 [file acmi-5-543.v3-s001.pdf]
